# Extraction of Anthocyanins from Borage (*Echium amoenum*) Flowers Using Choline Chloride and a Glycerol-Based, Deep Eutectic Solvent: Optimization, Antioxidant Activity, and In Vitro Bioavailability

**DOI:** 10.3390/molecules27010134

**Published:** 2021-12-27

**Authors:** Oscar Zannou, Hojjat Pashazadeh, Mohamed Ghellam, Salam A. Ibrahim, Ilkay Koca

**Affiliations:** 1 Food Engineering Department, Faculty of Engineering, Ondokuz Mayis University, Samsun 55139, Turkey; hojjat_pashazadeh@yahoo.com (H.P.); mohamed.gh2010@gmail.com (M.G.); itosun@omu.edu.tr (I.K.); 2 VetAgro-Sup, Agronomic Campus, 63370 Clermont-Ferrand, France; 3 Food and Nutritional Sciences Program, North Carolina A&T State University, Greensboro, NC 27411, USA; ibrah001@ncat.edu

**Keywords:** borage, *Echium amoenum*, phenolic compounds, antioxidant activity, bioavailability

## Abstract

Borage flower (*Echium amoenum*), an annual herb native to the Mediterranean region, is an excellent source of anthocyanins and is widely used in various forms due to its biological activities. In the present study, a choline chloride and glycerol (CHGLY)-based natural deep eutectic solvent (NADES) was applied in order to extract the anthocyanins from borage flowers. The traditional solvents, including water, methanol, and ethanol, were used to evaluate the efficiency of CHGLY. The results showed that CHGLY was highly efficient compared to the traditional solvents, providing the highest amounts of the total anthocyanin content (TAC), total phenolic content (TPC), total flavonoid content (TFC), individual anthocyanins, and antioxidant activity (DPPH radical scavenging (DPPH) and ferric-reducing antioxidant power (FRAP) assays). The most dominant anthocyanin found in studied borage was cyanidin-3-glucoside, followed by cyanin chloride, cyanidin-3-rutinoside, and pelargonidin-3-glucoside. The bioavailability % was 71.86 ± 0.47%, 77.29 ± 0.57%, 80.22 ± 0.65%, and 90.95 ± 1.01% for cyanidin-3-glucoside, cyanidin-3-rutinoside, by pelargonidin-3-glucoside and cyanin chloride, respectively. However, cyanidin-3-glucoside was the anthocyanin compound showing the highest stability (99.11 ± 1.66%) in the gastrointestinal environment. These results suggested that choline chloride and glycerol-based NADES is not only an efficient, eco-friendly solvent for the extraction of anthocyanins but can also be used to increase the bioavailability of anthocyanins.

## 1. Introduction

Human health and well-being are closely linked to one’s environment, diet, and overall lifestyle. Free radicals are associated with increased incidence of cardiovascular, pulmonary diseases, and many types of cancers [1]. Like reactive oxygen species (ROS) and reactive nitrogen species (RNS), free radicals can be produced in the organism as a by-product of metabolism or introduced from a number of exogenous sources (pollution, radiation, drugs, etc.) [2,3]. Free radicals can adversely affect many biological molecules (nucleic acids, proteins, and lipids), which alters the biological activities and results in increased oxidative stress. Consequently, they are involved directly or indirectly in the activation of diseases, such as diabetes, neurodegenerative disorders, respiratory diseases, cardiovascular diseases, along with other various diseases and cancers [2]. Through the ages, humans have believed in the positive effects associated with using a variety of herbs and foods for the treatment of certain illnesses. More recently, over the past few decades, researchers have begun to study the composition and purported effects of herbal treatments used in traditional medicine.

Borage or *Echium amoenum* is a member of the Boraginaceae family and one of the popular annual herbs used in traditional medicine in several countries. Borage is dispersed in many parts of Europe, the Mediterranean basin, and northern Iran. Its stems and leaves are hairy and bristly, and it has bright blue, star-shaped flowers, which are the most used part of the plant [4]. This plant has been used as a decoction separately or in combination with other herbs [5]. It has been traditionally used as a sedative, antidepressant, antifebrile, anti-inflammatory, for infectious diseases and influenza, and also used against cardiovascular and pulmonary diseases. Recently, it has been widely believed to have possible effects against various types of cancers [3,4]. Many recent in vivo, in vitro, and clinical studies have been conducted in an effort to prove the therapeutic effects of the borage plant. It was found that its flowers possess an antiviral [4], antioxidant [3,6], antibacterial activity against Gram-positive and Gram-negative bacteria [6,7], as well as anti-inflammatory effects [6,8]. It was also suggested that borage flowers could be a natural co-treatment due to their neuroprotective effects [9], anti-cancer proprieties and reduction of oxidative stresses [3,6], anxiolytic effect [10], and an inhibitory effect with regard to enzymes-related health issues [11]. 

Those positive effects are mostly attributed to the chemical composition, owing to the presence of phenolics (pyrogallol, salicylic acid, gallic acid, caffeic acid), flavonoids (rutin, myricetin), fatty acids (α-linolenic, palmitic, stearidonic) [6], and anthocyanins [5,12]. Borage also contains rosmarinic acid, which is one of the carboxylic acids believed to have beneficial effects on human health [11,13]. Besides, borage can be a good source of minerals (iron, calcium, phosphorus) and soluble and insoluble fibers [5]. Various studies have been carried out to assess the effect of treatments on the composition and biological activities of the borage plant. For example, Nadi et al. [14] investigated the effect of fluidized drying parameters (temperature, air velocity) on energy consumption and the quality (color, phenolic compounds, anthocyanins, antioxidant activity) of borage petals. Their results showed a decrease in energy consumption and preservation of the quality and maintenance of the bioactive components of the dried petals. Mehran et al. [12] evaluated the encapsulation process of anthocyanin extracts using spray drying and maltodextrin/modified starch. Their results indicated a high encapsulation efficiency and an increase in the stability of anthocyanin. 

In order to take advantage of their biochemical activities, the extraction of different bioactive compounds from the borage plant has been realized using a variety of solvents. The extracts obtained with various solvents exhibited different methods and amplitudes of the response. For instance, methanolic extracts of flowers possessed the highest antibacterial and antioxidant activities compared to the ethanolic and aqueous extracts [6]. In a previous study, it was found that the ethanolic extract of borage flowers exhibited a significantly higher antibacterial activity than the aqueous extracts [7]. In addition, the application of assistive techniques (e.g., pulsed electric field) has improved the efficiency of polyphenol extraction, and thus the antioxidant capacity of aqueous leaf extracts [15].

Recently, the application of natural deep eutectic solvents (NADESs) for the isolation of bioactive compounds has revolutionized their extraction processes. NADESs are a mix between the hydrogen bond acceptor (HBA) and hydrogen bond donor (HBD). NADESs have been found to be excellent solvents for separating and stabilizing phenolic compounds [16,17,18] because they form hydrogen bonds with phenolic compounds and increase solubility [19]. NADESs are highly efficient at extracting the phenolic compounds from various plant materials compared to extraction by traditional solvents such as methanol, ethanol, hexane, dichloromethane, and water [20,21,22,23].

To the best of our knowledge, no published studies investigating the anthocyanins from borage using NADESs have been found in the existing literature. In the present study, choline chloride and glycerol-based NADESs (CHGLY) were prepared and applied for the extraction of anthocyanins from borage. Water, ethanol, and methanol were used as conventional solvents for comparison. Moreover, total phenolic, total flavonoid, total anthocyanin contents, DPPH radical scavenging, and FRAP of borage extracts were evaluated. In addition, the extraction conditions with the prominent CHGLY were optimized using the central composite of Response Surface Methodology (RSM), and the in vitro bioavailability of the extract obtained at the optimum conditions was determined.

## 2. Results and Discussion

### 2.1. FTIR Spectra, Viscosity, pH, and Conductivity of CHGLY

**Figure 1:**
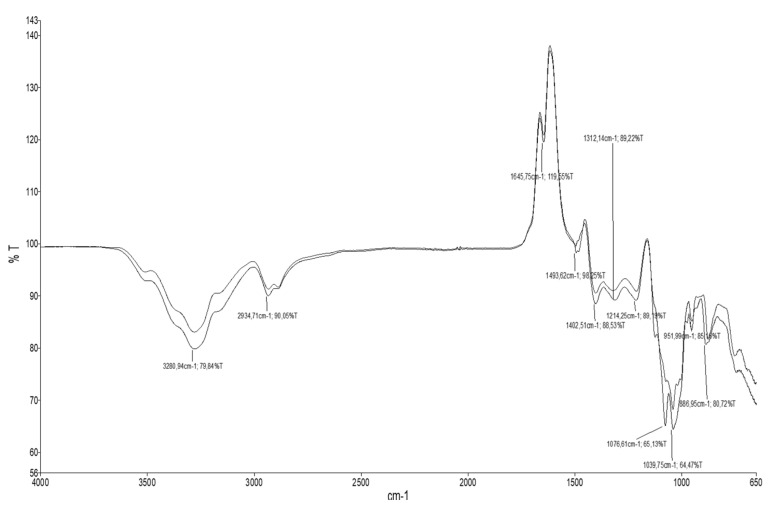
FTIR spectrum of natural deep solvent prepared from choline chloride and glycerol (CHGLY).

The FTIR spectrum of NADES obtained from choline and glycerol (CHGLY) is shown in Figure 1. The low intensity of OH stretching bands at wavenumber 3500–3200 cm^−1^ confirmed the presence of a low quantity of water in CHGLY [24,25]. During the CHGLY preparation, 20% of water was introduced to tailor the viscosity and to facilitate manipulation and enhance the extraction performance. The OH stretching vibration at the wavenumber of 3300–3100 cm^−1^ indicated the formation of hydrogen bonding between HBA and HBD [24,26]. The wavenumber at 3200–2932 cm^−1^ and 1645 cm^−1^ wavenumber referred to C-H stretching bands and C=C stretching vibrations, respectively. In addition, the wavenumbers 1500–600 cm^−1^ correspond to the C–O, CH, C–C, and OCO stretching, deformation, or bending vibrations [27]. Thus, the CHGLY can be assessed to be a homogenized NADES in which the chemical group tied different bunding networks.

The viscosity, pH, and electric conductivity of CHGLY were found to be 22.89 ± 0.10 mPa, 5.03 ± 0.01, and 770.50 ± 10.25 µS.cm^−1^, respectively. Viscosity is an important factor for the NADESs application in the extraction of bioactive compounds. NADES with high viscosity decreases the mass transfer in the extraction matrix [18]. In accordance with the viscosity found in the present study, Yadav et al. [28] have reported a viscosity of 21.37 mPa for the NADES prepared with choline chloride and glycerol at a 1:2 molar ratio and with the addition of 20% of water. Additionally, CHGLY has been reported as an adequate NADES for the extraction of phenolic compounds [29].

### 2.2. Screening CHGLY Efficiency vs. Conventional Solvents

**Figure 2:**
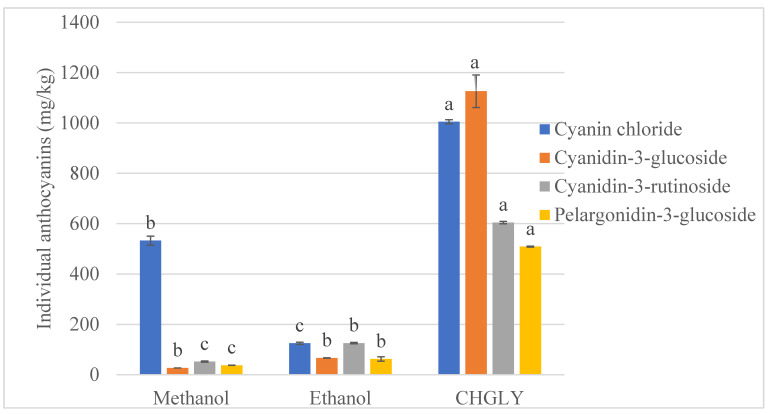
Individual anthocyanins extracted from borage using different solvents. a–c = Different lowercase letters on the bars with the same color indicate significant differences between solvents (*p* < 0.05).

The extraction efficiency of CHGLY compared to methanol, ethanol, and water was investigated, and the results are given in Table 1. The extractability of TAC, TPC, TFC, DPPH, and FRAP changed greatly depending on the type of solvents (*p* < 0.05). The TAC, TPC, TFC, DPPH, and FRAP determined in borage were found in the ranges 0.07–2.61 mg c3gE/g, 10.08–27.76 mg GAE/g, 2.34–10.29 mg ECE/g, 48.35–146.92 mmol TE/g, and 444.73–939.85 mmol ISE/g, respectively. Similar to our findings, Bekhradnia and Ebrahimzadeh [30] have determined TPC of 3.79–41.69 mg/g and TFC of 1.14–11.11 mg/g from various extracts of borage. In contrast, Asghari et al. [11] have reported higher values of TPC (90.20–296.20 mg/g) and TFC (48.40–115.90 mg/g) from different extracts of borage. These variations could be linked to the growing conditions of the raw material, extraction, and analysis conditions. In the present study, four anthocyanin compounds, including cyanin chloride, cyanidin-3-glucoside, cyanidin-3-rutinoside, and pelargonidin-3-glucoside, were detected in borage various extracts (Figure 2). The recovery of these anthocyanins changed greatly depending on the solvent used for the extraction (*p* < 0.05). The most dominant anthocyanin found in the studied borage was cyanidin-3-glucoside (26.97 ± 0.08–1126.45 ± 64.72 mg/kg), followed by cyanin chloride (532.65 ± 17.79–1005.01 ± 8.20 mg/kg), cyanidin-3-rutinoside (52.34 ± 2.12–604.36 ± 4.74 mg/kg), and pelargonidin-3-glucoside (37.71 ± 0.23–508.86 ± 2.05 mg/kg). Accordingly, cyanidin-3-glucoside has been reported as the most important anthocyanin in borage [12,31,32].

As can be observed in Table 1, CHGLY exhibited the highest performance of all evaluated phytochemical characteristics, followed by water, methanol, and ethanol. However, no anthocyanin was determined in the aqueous extract. Likewise, the highest amounts of all identified individual anthocyanins were found in the CHGLY extract. CHGLY was followed by methanol for cyanin chloride and by ethanol for cyanidin-3-rutinoside and pelargonidin-3-glucoside (Figure 2). Several studies have reported similar results in which different NADESs have been found to be more efficient than conventional solvents for the recovery of phenolic compounds, such as anthocyanins [19,21,33,34]. The highest efficiency of CHGLY could also be associated with multiple hydrogen bonding networks formed by the different chemical groups of choline chloride and glycerol [35,36], which interact and facilitate the extraction of phenolic compounds [37,38]. Moreover, phenolic compounds are polar molecules, and their extraction is prominent with highly polar solvents. Recently, polyalcohol-based NADESs, including CHGLY, have been reported amongst the most polar and efficient for the recovery of phenolic compounds plants [29,39,40]. Therefore, CHGLY was selected for further analyses and optimization of extraction conditions.

### 2.3. Optimization of Extraction Conditions 

The extraction optimization of anthocyanins from borage petals using deep eutectic solvents and applying a three-level central composite design was realized using four independent factors (X_1_; molar ratio, X_2_; water content, X_3_; temperature, and X_4_; extraction time). The results of different experimental points are shown in Table 2.

As can be seen in Table 2, the nine responses demonstrated different variations of the values of experimental data. For instance, the analyzed spectrophotometric responses, TAC, TPC, TFC, FRAP, and DPPH, ranged between 2.64 and 6.73 mg CGE/100 g, 26.96 and 53.20 mg GAE/g, 10.54 and 26.85 mg ECE/g, 505.77 and 972.43 mmol ISE/g, and 55.88 and 335.31 mmol TE/g, respectively. Except TAC, which exhibited a high extraction efficiency at run 17 (X_1_; 1:2 molar ratio, X_2_; 20% water content, X_3_; 40 °C, and X_4_; 35 min), the rest, TPC, TFC, FRAP, and DPPH, had their highest value at run 13 (X_1_; 1:3.5 molar ratio, X_2_; 30% water content, X_3_; 60 °C, and X_4_; 45 min). Meanwhile, the chromatographic analysis of individual anthocyanins had another trend. They widely ranged between 18.07 and 1554.68 mg/kg, 10.16 and 1798.48 mg/kg, 53.84 and 707.30 mg/kg, and 56.25 and 726.86 mg/kg, for cyanidin chloride, cyanidin-3-glucoside, cyanidin-3-rutinoside, and pelargonidin-3-glucoside, respectively. The best recovery of cyanidin chloride and cyanidin-3-glucoside was at run 26 (X_1_; 1:3.5 molar ratio, X_2_; 30% water content, X_3_; 60 °C, and X_4_; 5 min), cyanidin-3-rutinoside at run 10 (X_1_; 1:2 molar ratio, X_2_; 20% water content, X_3_; 75 °C, and X_4_; 35 min), and pelargonidin-3-glucoside at run 14 (X_1_; 1:3.5 molar ratio, X_2_; 30% water content, X_3_; 60 °C, and X_4_; 25 min). Apparently, except for TAC, high-temperature extraction (60–75 °C) seems to be efficient in recovering antioxidant compounds and increasing the antioxidant capacity of extracts. The molar ratio appears as a second influencing factor in a range between 2 and 3.5 of glycerol to 1 choline chloride molar ratio.

ANOVA obtained results are shown in Table 3. According to them, the quadratic model has been found suitable for the representation of experimental data. In general, and for all responses, the significance of the model was very low *(p* < 0.0001), and the insignificant lack-of-fit had high values (*p* > 0.1735). Additionally, the model had satisfactory R^2^ (>0.9429) and adjusted-R^2^ (>0.8762). All this confirms the closeness between experimental and the predicted values. After the model selection, the developed model terms of all the responses are shown in Table 3. Generally, temperature linear terms (X_3_) were highly significant (*p* < 0.0001) for most responses, except for FRAP and cyanidin-3-rutinoside responses. According to the number of responses and their significance, the temperature was followed by water content (X_2_), time (X_4_), and finally by molar ratio linear terms; however, time quadratic terms (X_4×4_) were highly significant (*p* < 0.0009) for all responses, excluding the cyanidin-3-rutinoside response. Molar ratio quadratic terms (X_1_X_1_) exhibited significance for many responses, followed by temperature (X_3_X_3_) and water content (X_2_X_2_) quadratic terms. Moreover, terms of interactions have shown another tendency, where molar ratio–time (X_1_X_4_) interactions had a high significance for most responses (*p* < 0.0343), followed by temperature–time (X_3_X_4_), temperature–water content (X_2_X_3_), and time–water content (X_2_X_4_) interactions. The final polynomial equations are given in terms of the coded factors for all studied responses as follows:

(1) $$\begin{array}{cc} \mathrm{TAC}\;\mathrm{mg}\;\mathrm{CGE}/100\;g & =3.33-0.07X_{1}+0.13X_{2}-0.12{\;X}_{3}+0.18X_{4}+0.15X_{1}{\;X}_{2}+0.60X_{1}{\;X}_{3}-0.36{\;X}_{1}{\;X}_{4}\\ & +0.11X_{2}{\;X}_{3}-0.33X_{2}{\;X}_{4}-0.54X_{3}{\;X}_{4}+0.21X_{1}{\;X}_{1}-0.07X_{2}{\;X}_{2}+0.26X_{3}{\;X}_{3}+0.27X_{4}{\;X}_{4} \end{array}$$

(2) $$\begin{array}{cc} \mathrm{TPC}\;\mathrm{mg}\frac{\mathrm{GAE}}{g} & =40.46-0.27X_{1}+2.72{\;X}_{2}+3.47{\;X}_{3}+1.80{\;X}_{4}-1.12{\;X}_{1}{\;X}_{2}-0.39{\;X}_{1}{\;X}_{3}-0.66{\;X}_{1}{\;X}_{4}\\ & -0.49{\;X}_{2}{\;X}_{3}-1.01{\;X}_{2}{\;X}_{4}-1.00{\;X}_{3}{\;X}_{4}-1.54{\;X}_{1}{\;X}_{1}-1.80{\;X}_{2}{\;X}_{2}-0.66{\;X}_{3}{\;X}_{3}\\ & +2.09X_{4}{\;X}_{4} \end{array}$$

(3) $$\begin{array}{cc} \mathrm{TFC}\;\mathrm{mg}\frac{\mathrm{ECE}}{g} & =15.44+0.51{\;X}_{1}+1.08{\;X}_{2}+2.68{\;X}_{3}+0.50X_{4}-0.57X_{1}{\;X}_{2}+0.76{\;X}_{1}{\;X}_{3}+0.64{\;X}_{1}{\;X}_{4}\\ & -0.53{\;X}_{2}{\;X}_{3}+1.77{\;X}_{2}{\;X}_{4}-1.21{\;X}_{3}{\;X}_{4}-0.85{\;X}_{1}{\;X}_{1}-0.34{\;X}_{2}{\;X}_{2}+0.51{\;X}_{3}{\;X}_{3}\\ & +2.43{\;X}_{4}{\;X}_{4} \end{array}$$

(4) $$\begin{array}{cc} \mathrm{FRAP}\;\mathrm{mmol}\frac{\mathrm{ISE}}{g} & =717.70+20.46{\;X}_{1}+40.53{\;X}_{2}+7.37{\;X}_{3}+2.49{\;X}_{4}-30.27X_{1}{\;X}_{2}+8.98{\;X}_{1}{\;X}_{3}\\ & +48.71{\;X}_{1}{\;X}_{4}-35.30{\;X}_{2}{\;X}_{3}+10.70{\;X}_{2}{\;X}_{4}+24.74{\;X}_{3}{\;X}_{4}-0.004{\;X}_{1}{\;X}_{1}-22.09{\;X}_{2}{\;X}_{2}\\ & -11.14{\;X}_{3}{\;X}_{3}+50.66{\;X}_{4}{\;X}_{4} \end{array}$$

(5) $$\begin{array}{cc} \mathrm{DPPH}\;\mathrm{mmol}\frac{\mathrm{TE}}{g} & =208.97+7.15X_{1}-8.56X_{2}+23.56X_{3}+0.55X_{4}-8.78X_{1}{\;X}_{2}-19.58{\;X}_{1}{\;X}_{3}-21.74{\;X}_{1}{\;X}_{4}\\ & +1.20{\;X}_{2}{\;X}_{3}+0.93{\;X}_{2}{\;X}_{4}+2.59{\;X}_{3}{\;X}_{4}-34.98{\;X}_{1}{\;X}_{1}+4.74X_{2}{\;X}_{2}+17.29X_{3}{\;X}_{3}\\ & +27.41X_{4}{\;X}_{4} \end{array}$$

(6) $$\begin{array}{cc} \mathrm{Cyanidin}\;\mathrm{chloride}\frac{\mathrm{mg}}{\mathrm{kg}} & =418.45+10.15X_{1}+97.59{\;X}_{2}+185.00{\;X}_{3}-104.15{\;X}_{4}+60.02{\;X}_{1}{\;X}_{2}+12.06X_{1}{\;X}_{3}\\ & +60.27{\;X}_{1}{\;X}_{4}-190.77X_{2}{\;X}_{3}+11.55{\;X}_{2}{\;X}_{4}+18.34{\;X}_{3}{\;X}_{4}+79.86X_{1}{\;X}_{1}+19.25{\;X}_{2}{\;X}_{2}\\ & -8.60{\;X}_{3}{\;X}_{3}+250.42{\;X}_{4}{\;X}_{4} \end{array}$$

(7) $$\begin{array}{cc} \mathrm{Cyanidin}-3-\mathrm{glucoside}\frac{\mathrm{mg}}{\mathrm{kg}} & =599.31+60.96{\;X}_{1}+87.39{\;X}_{2}+251.45{\;X}_{3}-195.38X_{4}-132.42{\;X}_{1}{\;X}_{2}+32.53{\;X}_{1}{\;X}_{3}\\ & -185.95X_{1}{\;X}_{4}-38.34{\;X}_{2}{\;X}_{3}+22.47X_{2}{\;X}_{4}+165.88{\;X}_{3}{\;X}_{4}+135.84{\;X}_{1}{\;X}_{1}-5.14{\;X}_{2}{\;X}_{2}\\ & -27.63X_{3}{\;X}_{3}+226.00{\;X}_{4}{\;X}_{4} \end{array}$$

(8) $$\begin{array}{cc} \mathrm{Cyanidin}-3-\mathrm{rutinoside}\frac{\mathrm{mg}}{\mathrm{kg}} & =458.98-11.44{\;X}_{1}-35.11{\;X}_{2}-14.44{\;X}_{3}-24.86{\;X}_{4}+76.75{\;X}_{1}{\;X}_{2}-24.96{\;X}_{1}{\;X}_{3}\\ & -66.95X_{1}{\;X}_{4}-131.89{\;X}_{2}{\;X}_{3}-43.96{\;X}_{2}{\;X}_{4}-9.28{\;X}_{3}{\;X}_{4}-18.59{\;X}_{1}{\;X}_{1}+12.27{\;X}_{2}{\;X}_{2}\\ & -71.07{\;X}_{3}{\;X}_{3}-21.41{\;X}_{4}{\;X}_{4} \end{array}$$

(9) $$\begin{array}{cc} \mathrm{Pelargonidin}-3-\mathrm{glucoside}\frac{\mathrm{mg}}{\mathrm{kg}} & =643.07-71.75X_{1}-2.38{\;X}_{2}-87.24{\;X}_{3}\mp 38.18{\;X}_{4}-5.77{\;X}_{1}{\;X}_{2}+48.22{\;X}_{1}{\;X}_{3}\\ & -20.28{\;X}_{1}{\;X}_{4}-102.87{\;X}_{2}{\;X}_{3}-49.76X_{2}{\;X}_{4}+72.04X_{3}{\;X}_{4}-80.32X_{1}{\;X}_{1}-33.32{\;X}_{2}{\;X}_{2}\\ & -97.16{\;X}_{3}{\;X}_{3}-91.75{\;X}_{4}{\;X}_{4} \end{array}$$

**Figure 3:**
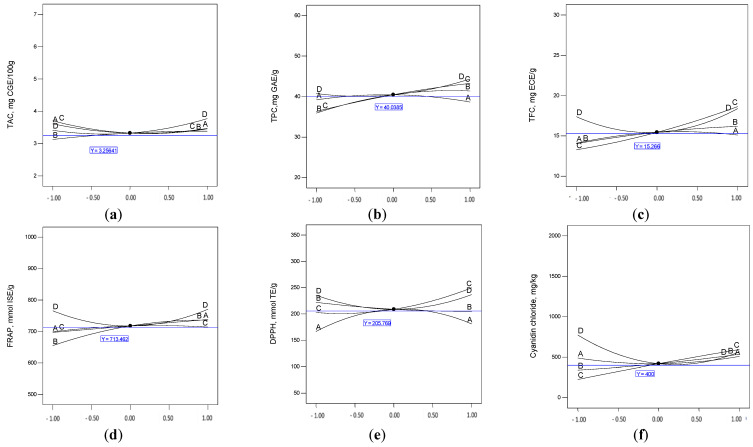

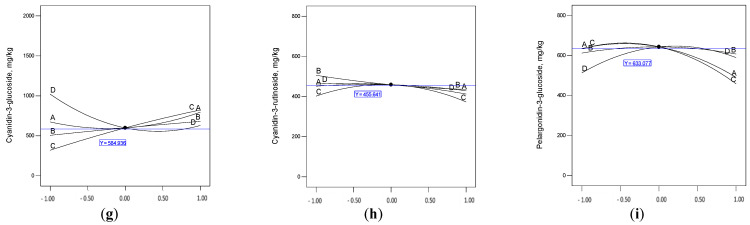
Perturbation plots showing the effects of the fours experimental factors (A: molar ratio, B: water content, C: temperature, D: time) on the analyzed responses ((**a**): TAC, (**b**): TPC, (**c**): TFC, (**d**): FRAP, (**e**): DPPH, (**f**): cyanidin chloride, (**g**): cyanidin-3-glucoside, (**h**): cyanidin-3-rutinoside, and (**i**): pelargonidin-3-glucoside).

Model and terms are significant at *p* ≤ 0.05.The previous equations are used to generate the perturbation plots (Figure 3), which helps to compare the effect of factors in one chosen point (A = 3.5, B = 30, C = 58, D = 25). A, C, B, and D represent molar ratio, water content, temperature, and time, respectively. For TAC (Figure 3a), factors, the molar ratio (A), temperature (C), and time (D) had a high extraction effect at their low and high limits and gave a weak extraction at the center point. However, water content factor (B) gave the lowest values in low limits, which means that the extraction efficiency of TAC was very weak at low water content, and after that, the effect was stabilized. Time seemed to be more effective more than other factors for long-duration treatment.

Moreover, TPC demonstrated another tendency (Figure 3b); the extraction of phenolic compounds has a positive relationship with water content and temperature; meanwhile, the molar ratio gives a high extraction efficiency when it approaches from the center point. Time presented more efficiency after 25 min of extraction. Similar to TPC, TFC extraction efficiency had a positive relationship with increasing temperature. FRAP analysis presented the effect of increasing water content and almost a stable effect of molar ratio and temperature variation. The antiradical scavenging capacity presented by DPPH (Figure 3e) has been affected; positively by increasing extraction temperature and negatively by increasing water content. Varying the molar ratio was more efficient around the midpoint. Beyond the center point, extraction time was more efficient at positive and negative extremes to give the highest values for TFC, FRAP, and DPPH.

However, the long extraction time affected the content of most individual anthocyanins (Figure 3f–i). Unexpectedly, a short extraction time gave a high content of cyanidin chloride and cyanidin-3-glucoside, more than 750 mg/kg and 1000 mg/kg.b respectively. Meanwhile, for the same components, the high levels of molar ratio, water content, and temperature enhanced their extraction. For almost all factors, high levels negatively affected the extraction of cyanidin-3-rutinoside. The high molar ratio and extraction temperature drastically decreased the content of recovered pelargonidin-3-glucoside; in contrast, a short extraction time also gave weak levels.

The obtained results are consistent with a list of studies performed on the effect of extraction conditions using deep eutectic solvents. For instance, the study of de Almeida Pontes et al. [41] on olive leaves showed an improvement in the extraction of phenolic compounds by increasing extraction temperature (>50 °C). Additionally, it was demonstrated that variation in the amount and composition of deep eutectic solvents contents influences the composition and the quantity of recovery phenolic compounds. In addition, Cui et al. [42] found that the extraction yield of polyphenols was closely related to extractions parameters (time, temperature, liquid ratio, water content). Da Silva et al. [25] analyzed the effect of the molar ratio of choline-chloride: glycerol: citric acid mixture on the extraction of blueberry anthocyanins. They indicated the existence of a relationship between the anthocyanin’s extraction efficiency and the chosen molar ratio. In the same way, Zannou et al. [21] observed that deep eutectic extraction behaves differently, and anthocyanins were sensible to all the studied factors (molar ratio, solvent ratio, and additional water).

### 2.4. Multi-Response Optimization on the Responses Using RSM

**Figure 4:**
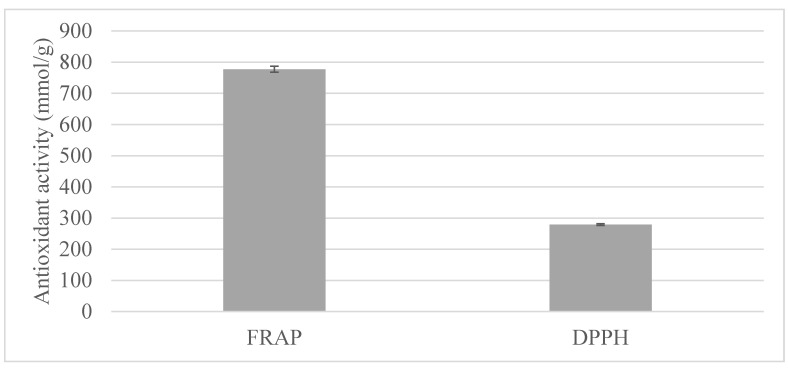
Antioxidant activity of borage (*Echium amoenum*) flowers obtained in the optimum conditions.

**Figure 5:**
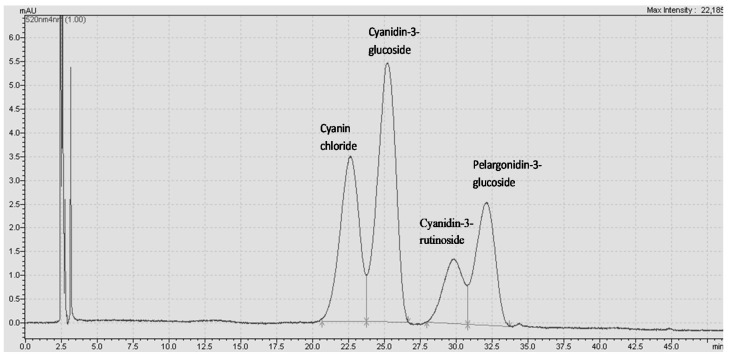
HPLC chromatogram of individual anthocyanins obtained fron borage flowers under optimal conditions.

RSM was performed to identify the optimum conditions to obtain the maximum responses. The optimum conditions were determined by applying the desirability function, where the independent variables were kept in range, and the responses were maximized. The optimum conditions for maximum responses were a 1:4.62 molar ratio, 23.33% water content, a temperature of 74 °C, and 15 min extraction time. Under these optimum conditions, the predicted values of TAC, TPC, TFC, FRAP, DPPH, cyanin chloride, cyanidin-3-glucoside, cyanidin-3-rutinoside, and pelargonidin-3-glucoside were 4.39 mg c3gE/g, 42.65 mg GAE/g, 22.72 mg ECE/g, 731.87 mmol ISE, 273.48 mmol TE/g, 995.74 mg/kg, 1409.78 mg/kg, 447.85 mg/kg, and 234.38 mg/kg, respectively. Further analyses were conducted in triplicate under the same optimum conditions to confirm the predicted data. The experimental results were found as 4.37 ± 0.34 c3gE/g, 54.96 ± 5.23 mg GAE/g, 28.85 ± 0.85 mg ECE/g, 777.38 ± 9.64 mmol ISE/g, 279.13 ± 2.96 mmol TE/g, 1085.37 ± 11.49 mg/kg, 1418.91 ± 7.71 mg/kg, 448.01 ± 5.22 mg/kg, and 299.39 ± 0.97 mg/kg for TAC, TPC, TFC, FRAP, DPPH, cyanin chloride, cyanidin-3-glucoside, cyanidin-3-rutinoside, and pelargonidin-3-glucoside. The predicted and experimental data were found to be very close, which confirmed the reliability and reproducibility of the applied RSM process. As can be observed in Figure 4, the extract obtained in the optimum conditions had a high antioxidant activity, with the FRAP providing the highest value. Also. Figure 5 shows the chromatogram of individual anthocyanins obtained under optimal conditions.

### 2.5. In Vitro Bioavailability

**Figure 6:**
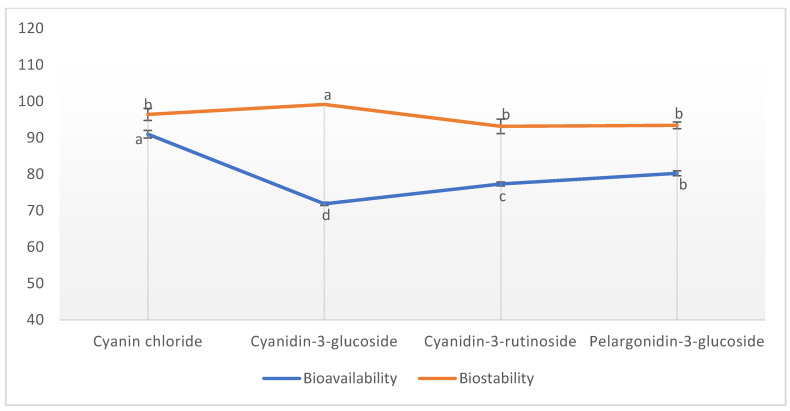
Bioavailability and stability of the individual anthocyanins extracted from borage using CHGLY. a–d = Different lowercase letters on the graphics indicate significant differences (*p* < 0.05).

An in vitro gastrointestinal model was applied to mimic the different steps of in vivo physiological digestion. Anthocyanins are the type of pH-sensitive phenolic compounds present in different chemical structures. The main chemical forms are flavylium cations in the stomach, while the carbinol forms predominate in the intestinal environment [43]. The bioavailability % and biostability % of the anthocyanin-enriched extract were investigated considering cyanin chloride, cyanidin-3-glucoside, cyanidin-3-rutinoside, and pelargonidin-3-glucoside (Figure 6). The bioavailability of the evaluated anthocyanin compounds varied greatly in the simulated intestinal digestion (*p* ≤ 0.05). After the simulated intestinal digestion, cyanin chloride exhibited the highest bioavailability (90.95 ± 1.01%), followed by pelargonidin-3-glucoside (80.22 ± 0.65%), cyanidin-3-rutinoside (77.29 ± 0.57%), and cyanidin-3-glucoside (71.86 ± 0.47%), respectively. These findings were found close to the previous results of Mehran et al. [12], who determined a range of bioavailability of 70–90% for the anthocyanin extract of borage. Furthermore, Oliveira and Pintado [44] and Koh et al. [45] reported 88% and 90% bioavailability of cyanidin-3-glucoside after simulated intestinal digestion. Generally, anthocyanins are destroyed or biotransformed into other substances in the intestinal environment due to the high pH. Nonetheless, the bioavailability found in the present study was high and ranged from 70 to 90%, suggesting that CHGLY exerted a protective effect on borage anthocyanins. Anthocyanin compounds were less degraded due to the strong hydrogen bunding formed between CHGLY and anthocyanins. Similar to our findings, Da Silva et al. [46] found that the intestinal bioaccessibility of phenolic compounds was remarkably increased in the extract obtained from NADES (choline chloride:glycerol:citric acid; 0.5:2:0.5 molar ratio) compared to the extract obtained from conventional organic solvent (methanol:water:formic acid; 50:48.5:1.5; *v*/*v*/*v*), being about 35-fold higher for anthocyanins and 5-fold higher for non-anthocyanin phenolic compounds. Furthermore, Huang et al. [47] concluded that NADES is not only a sustainable ionic liquid with higher extraction efficiency but also an enhancer of oral bioavailability of specific natural products. The biotransformation of anthocyanins during the gastrointestinal tract changes greatly during the phase II metabolism processes and enzymatic and microbiota catabolism [43,48]. Di Lorenzo et al. [43] determined that the food matrix or technological/processing conditions, enzymatic patterns, and microbiota composition are the main factors affecting the bioavailability of anthocyanins in the gastrointestinal environment. As shown in Figure 6, the stability of the evaluated anthocyanins was found to be different in the intestinal environment. Although cyanidin-3-glucoside exhibited a low bioavailability compared to other anthocyanins, it presented the highest stability (99.11 ± 1.66%) in the intestinal environment. Cyanidin-3-glucoside was followed by cyanin chloride (96.37 ± 1.66%), pelargonidin-3-glucoside (93.39 ± 0.93%), and cyanidin-3-rutinoside (93.13 ± 1.96%).

## 3. Material and Methods

### 3.1. Plant Material

**Figure 7:**
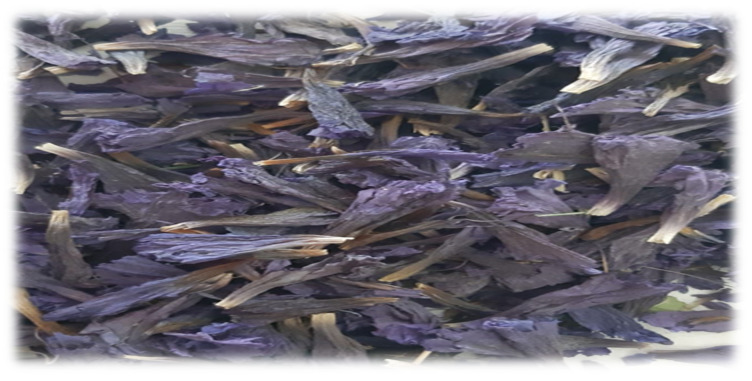
Photograph of the shade-dried borage (*Echium amoenum*) flowers.

Borage (*Echium amoenum*) flowers (Figure 7) were collected in August 2021 from Oroumieh, Iran. The flowers were shade-dried for five days, sorted, and packed in brown bottles’ screw caps.

### 3.2. Chemical and Reagents

Folin–Ciocalteu’s reagent, 2,2-diphenyl-1-picrylhydrazyl (DPPH), methanol (HPLC grade), ethanol (HPLC grade), acetonitrile (HPLC grade), sodium carbonate, sodium acetate, sodium nitrite, sodium hydroxide, hydrochloric acid (37%), choline chloride (≥98%), and standards were purchased from Sigma-Aldrich Chemical Co. (St. Louis, MO). Gallic acid, glacial acetic acid (≥99.5%), and iron sulfate were purchased from Carlo Erba. Aluminum chloride and iron chloride were acquired from Merck, while glycerol (≥99.5%) was acquired from Tekkim.

### 3.3. Preparation of NADES

NADES was prepared according to Chanioti and Tzia [29]. HBA (Choline chloride) and HBD (glycerol) were combined at 1:2 molar ratio, followed by the addition of 20% of distilled water. Afterward, the mixture was heated for 2 h 30 min at 80 °C to obtain a homogenized liquid, briefly abbreviated CHGLY.

### 3.4. Physico-Chemical Characteristics of NADES 

Viscosity of CHGLY was determined at 30 °C using a Rheometer (Buchi, CH-9230 Flawil, Switzerland) fitted with a parallel geometry with 20 mm of diameter and gap 1 mm [22]. pH was measured using a pH-meter (Model Starter 3100, OHAUS, Parsippany, NJ, USA). FTIR analysis of NADESs and extracts was carried out at the wavenumbers of 4000 and 400 cm^−1^ using an FTIR Spectrometer (Perkin Elmer, Spectrum-Two, Watham, MA, USA, PEService 35) [21]. Electrical conductivity properties were measured using an electrochemical analyzer (Consort, c6010, Turnhout, Belgium). The measurements were performed at 25 °C, and the values were recorded as µS.cm^−1^.

### 3.5. Extraction of Anthocyanins 

The extraction was carried out using a water bath. Distilled water, methanol, and ethanol (conventional solvents), and CHGLY (NADES), were used as solvents. A total of 0.3 g was mixed with 10 mL of solvents, and the mixture was ultrasonicated at 25 °C for 20 min. The samples were then filtered through Whatman filter paper No.1 thrice.

### 3.6. Total Phenolic Content (TPC)

TPC was evaluated by Folin–Ciocalteu method adopted from Nguyen et al. [49] with some modifications. Briefly, 150 µL of samples were mixed with 750 µL of 10% Folin–Ciocalteu reagent (5 min) and 600 μL of 7.5% Na_2_CO_3_. The mixture was kept in dark for 2 h, and the absorbance was read at 760 nm. TPC was expressed as mg gallic acid equivalent per g (mg GAE/g).

### 3.7. Total Flavonoid Content (TFC)

TFC was determined by adopting the procedure mentioned in Kim et al. [50]. The absorbance was read at 510 nm. The results were given as mg epicatechin equivalent per g (mg ECE/g).

### 3.8. Total Anthocyanin Content (TAC)

TAC was determined with the pH differential method reported in Lee et al. [51]. The absorbances of the samples containing pH 1 and pH 4.5 were read at 510 and 700 nm. TAC was expressed as mg cyanidin-3-glucoside equivalent per 100 g (mg CGE/100 g).

### 3.9. Determination of Antioxidant Activity

#### 3.9.1. DPPH Radical Scavenging Activity Assay (DPPH)

DPPH assay was conducted following the method of Pashazadeh et al. [52]. The absorbance was read against a control. The values of DPPH radical scavenging were determined with a calibration curve as mmol Trolox equivalent per g (mmol TE/g).

#### 3.9.2. Ferric-Reducing Antioxidant Power Assay (FRAP)

FRAP assay was conducted following the method of Özdemir et al. [53]. The value of FRAP was obtained from a standard curve of FeSO_4_. The results were given as mmol FeSO_4_ equivalents per g (mmol ISE/g).

### 3.10. Determination of Individual Anthocyanins

The individual anthocyanins were identified using the previous method of Bosiljkov et al. [54] with modifications. The anthocyanins were determined using a high-pressure liquid chromatography (HPLC) system (Agilent 1260; Agilent Technologies) with a diode array detector (DAD) at 520 nm wavelength. The anthocyanins were separated in an Inertsil ODS-4 column (3 µm, 4.6 × 50 mm; GL Sciences Kat No: 5020-0404) at a 1 mL.min^−1^ flow rate. The mobile phases were: (A) 94% 2 mM sodium acetate and 6% acetic acid (*v*/*v*); and (B) acetonitrile. The following elution gradient was used, according to solvent B: 0–20 min, 14–23%; 20–40 min, 23–35%; 40–50 min, 40%; 50–60 min, 60%; 60–65 min 95%. The column temperature was set at 30 °C. The individual anthocyanins were identified by comparing their retention times with their respective standard. The identified anthocyanins were quantified using a mixture of external standards (cyanidin-3-glucoside, cyanidin-3-rutinoside, cyanidin chloride, and pelargonidin-3-glucoside), which were prepared at different concentrations. 

### 3.11. Optimization with Response Surface Method (RSM) 

The optimization parameters were examined systematically using response surface methodology based on the three-level central composite design (Design Expert software 13.0). The experimental design included four independent variables of X_1_ (CHGLY, molar ratio), X_2_ (water content, %), X_3_ (temperature, °C), and X_4_ (extraction time, min). The actual and coded values of the independent variables are shown in Table 4. The combination of parameters, such as molar ratio of CHGLY (1:0.5, 2, 3.5, 5, and 6.5), water content (10, 20, 30, 40, and 50%), temperature (25, 40, 60, 75, and 90 °C), and extraction time (5, 15, 25, 35, and 45 min), were chosen as independent variables. From these variables, RSM generated 27 experimental points, including three replicates at the central point. TPC, TFC, TAC, DPPH radical scavenging activity, FRAP, and individual anthocyanin compounds were chosen as the responses (Y). The experimental points, together with responses, are given in Table 2. The analyses were performed in triplicate, and the results were given as means ± standard deviation. The experimental data were fitted to the following quadratic polynomial model:

(10) $$Y=\beta_{0}+\overset{k}{\underset{i=1}{\sum }}\beta_{i}X_{i}+\overset{k}{\underset{i=1}{\sum }}\beta_{ii}X_{ii}+\overset{k-1}{\underset{i=1}{\sum }}\overset{k}{\underset{i=i+1}{\sum }}\beta_{ij}X_{i}X_{j}+Ƹ$$

where *Y* is the response; *X* is the independent variable; *β*_0_ is the model intercept coefficient; *β_i_*, *β_ii_*, *β_ij_* are interaction coefficients; *k* is the number of independent factors; and Ƹ is the experimental error. The relationship between independent variables and responses was examined using analysis of variance (ANOVA) test in the Design Expert program.

### 3.12. In Vitro Bioavailability

The in vitro bioaccessibility of anthocyanins was determined to be the fraction of anthocyanins that was solubilized within the mixed micelles and which became accessible for intestinal adsorption [55]. Following in vitro digestion, an aliquot of raw digesta was collected after the simulated small intestine digestion and centrifuged at 5000× *g* for 15 min at 4 °C. A supernatant (micelle fraction) was collected from the centrifuged digesta in which the anthocyanins were solubilized. A portion (3 mL) of the micelle fraction was vortexed after adding 3 mL of methanol and centrifuged at 5000× *g* for 15 min at 25 °C. The supernatant was then carefully collected and used for the determination of anthocyanins using HPLC-DAD. The bioaccessibility and stability of anthocyanins were then determined using the following equations:

Bioaccessibility (%) = (C_Micelle_/C_Digesta_) × 100 (11)

Stability (%) = (C_Digesta_/C_Initial_) × 100 (12)

where C_Initial_, C_Micelle_, and C_Digesta_ are the concentration of the individual anthocyanins initially, in the micelle phase, and the overall digesta at the end of the in vitro digestion, respectively.

### 3.13. Statistical Analyses 

All results were expressed as the mean of three replicates ± standard deviation. Statistical analyses were performed using a one-way analysis of variance ANOVA, and the significance of the difference between means was evaluated by Turkey’s test. Statistical significance was determined at *p* < 0.05. Design Expert software (version 13.0, Stat-Ease Inc., Minneapolis, MN, USA) was used for the RSM and experimental data analysis. ANOVA was used to determine the statistical relationship between factors. The adequacy of the models was determined by R^2^, adjusted R^2^, predicted R^2^, coefficient of variation (CV), adequate precision, *p*-value, and the value of Fisher’s test (F-value). The significance of the models and regression coefficients were measured at *p* < 0.05. The behaviors of variables and responses were checked by the perturbation graphics. The optimum conditions were determined by applying the desirability function.

## 4. Conclusions

In the present study, the extraction efficiency of borage anthocyanins was investigated using green choline chloride and glycerol-based NADES (CHGLY). The results revealed that the CHGLY was a promising and efficient medium for the recovery of borage anthocyanins. The use of CHGLY, water, methanol, and ethanol for the extraction of antioxidants from borage displayed the following ranges, 0.07–2.61 mg c3gE/g, 10.08–27.76 mg GAE/g, 2.34–10.29 mg ECE/g, 48.35–146.92 mmol TE/g, and 444.73–939.85 mmol ISE/g for TAC, TPC, TFC, DPPH, and FRAP, respectively. Four individual anthocyanins, cyanidin-3-glucoside, cyanin chloride, cyanidin-3-rutinoside, and pelargonidin-3-glucoside, were identified from borage extracts. The results revealed that that CHGLY performed better than traditional solvents and provided the highest amounts of total anthocyanin content (TAC), total phenolic content (TPC), total flavonoid content (TFC), and individual anthocyanins and antioxidant activity (DPPH and FRAP assays). The highest bioavailability was found with cyanin chloride (90.95 ± 1.01%), followed by pelargonidin-3-glucoside (80.22 ± 0.65%), cyanidin-3-rutinoside (77.29 ± 0.57%), and cyanidin-3-glucoside (71.86 ± 0.47%), respectively. The greatest biostability was found with cyanidin-3-glucoside, followed by cyanin chloride (96.37 ± 1.66%), pelargonidin-3-glucoside (93.39 ± 0.93%), and cyanidin-3-rutinoside (93.13 ± 1.96%). These findings suggest that CHGLY is a promising eco-friendly solvent that could be used as a sustainable, highly efficient, and green method for the extraction of bioactive plant compounds.

## Figures and Tables

**Table 1:** Antioxidant properties of borage extracts obtained from different solvents.

Solvents	Phytochemical Characteristics				
	TAC	TPC	TFC	DPPH	FRAP
CHGLY	2.61 ± 0.28 a	27.76 ± 0.31 a	10.29 ± 0.80 a	146.92 ± 11.84 a	939.85 ± 16.35 a
Ethanol	0.07 ± 0.03 c	10.08 ± 0.80 c	2.34 ± 0.44 c	48.35 ± 5.26 b	444.73 ± 1.52 d
Water	n.d.	26.50 ± 0.42 a	5.36 ± 0.36 b	124.61 ± 10.52 a	665.53 ± 0.00 b
Methanol	1.41 ± 0.11 b	20.33 ± 2.14 b	8.29 ± 0.26 a	120.89 ± 3.95 a	482.81 ± 6.83 c

a–d = Different lowercase letters indicate significant differences between solvents (*p* < 0.05); n.d. = not determined; TAC (mg c3gE); TPC (mg GAE/g); TFC (mg ECE/g); DPPH (mmol TE/g); FRAP (mmol ISE/g).

**Table 2:** Independent factors (X_1_, X_2_, X_3_, and X_4_) and experimental results for the responses.

Run	X_1_	X_2_	X_3_	X_4_	TAC	TPC	TFC	FRAP	DPPH	Cyanin Chloride	Cyanidin-3-Glucoside	Cyanidin-3-Rutinoside	Pelargonidin-3-Glucoside
1	5	40	40	35	3.92	38.44	18.90	795.47	189.52	860.61	10.16	410.84	237.87
2	5	40	75	35	4.06	45.41	23.06	824.8	214.17	834.99	968.08	53.84	137.55
3	2	40	75	15	3.99	43.11	18.93	758.02	253.30	971.10	1215.04	127.43	56.25
4	3.5	30	90	25	4.08	47.93	25.01	707.33	332.58	706.11	879.72	123.73	68.64
5	3.5	10	60	25	3.11	28.95	12.85	571.98	245.81	406.11	479.72	607.72	510.31
6	5	40	40	15	3.40	35.55	10.54	782.19	240.55	1005.80	1273.19	651.01	507.41
7	2	20	40	15	3.30	26.96	12.80	703.36	148.52	551.66	677.73	174.02	374.54
8	5	20	75	15	4.29	40.44	22.80	748.41	280.64	1166.77	1726.42	397.66	160.64
9	2	40	40	35	5.69	45.56	20.39	755.92	189.52	507.78	650.27	410.46	599.86
10	2	20	75	35	3.55	39.03	14.65	622.43	285.88	879.05	1062.68	707.30	523.18
11	3.5	30	60	25	3.40	39.74	17.36	680.24	215.76	495.32	751.63	480.66	534.20
12	5	20	75	35	3.64	37.55	18.19	840.3	218.68	956.59	1092.72	380.74	514.11
13	3.5	30	60	45	4.38	53.20	26.85	972.43	335.31	1377.59	1327.59	330.61	405.41
14	3.5	30	60	25	3.02	40.22	16.80	752.76	217.79	362.74	493.87	465.40	726,86
15	5	40	75	15	5.83	40.44	18.88	646.61	229.61	928.25	1340.97	375.35	83.83
16	0.5	30	60	25	4.14	36.36	11.80	683.39	55.88	723.11	990.96	439.36	486.50
17	2	20	40	35	6.73	32.14	11.57	505.77	205.01	101.39	50.27	371.56	416.50
18	2	40	40	15	4.06	34.51	14.13	885.05	167.65	970.69	960.69	330.67	692.36
19	2	20	75	15	2.76	39.18	20.21	708.62	242.39	1241.75	763.34	579.17	220.59
20	5	20	40	15	2.64	32.29	12.54	650.81	291.58	155.38	1080.62	262.85	236.88
21	3.5	30	25	25	4.75	27.61	11.36	652.91	216.86	100.89	140.72	245.00	446.71
22	3.5	50	60	25	3.02	38.66	17.31	703.24	207.93	676.81	797.21	434.45	506.93
23	2	40	75	35	3.08	45.96	18.65	729.64	277.00	696.74	1508.97	68.50	181.60
24	5	20	40	35	3.76	35.70	14.08	705.47	217.77	18.07	30.45	116,73	152,115
25	3.5	30	60	25	3.52	42,66	13.01	720.18	204.22	459.71	637.24	417.58	627.67
26	3.5	30	60	5	4.43	45.52	25.47	884.78	299.75	1554.68	1798.48	442.14	144.36
27	6.5	30	60	25	4.25	33.32	14.29	768.53	80.13	844.71	1413.81	355.91	154.76

TAC (mg c3gE); TPC (mg GAE/g); TFC (mg ECE/g); DPPH (mmol TE/g); FRAP (mmol ISE/g); cyanin chloride (mg/kg); cyanidin-3-glucoside (mg/kg); cyanidin-3-rutinoside (mg/kg); pelargonidin-3-glucoside.

**Table 3:** Results of ANOVA of the reduced models giving significant and non-significant terms.

	**TAC**			**TPC**			**TFC**		
	**SS**	**F-Value**	***p*-Value**	**SS**	**F-Value**	***p*-Value**	**SS**	**F-Value**	***p*-Value**
Model	22.46	19.25	<0.0001	984.14	14.15	<0.0001	543.53	17.56	<0.0001
X_1_	0.12	1.47	0.2489	1.77	0.3569	0.5613	6.26	2.83	0.1182
X_2_	0.40	4.83	0.0484	177.97	35.82	<0.0001	27.78	12.57	0.0040
X_3_	0.42	5.09	0.0436	325.83	65.58	<0.0001	194.39	87.95	<0.0001
X_4_	0.78	9.32	0.0100	77.57	15.61	0.0019	6.01	2.72	0.1250
X_1_ X_2_	0.36	4.37	0.0585	20.16	4.06	0.0669	5.18	2.34	0.1518
X_1_ X_3_	6.95	83.42	<0.0001	2.87	0.5779	0.4618	11.17	5.06	0.0441
X_1_ X_4_	2.05	24.60	0.0003	6.96	1.40	0.2595	6.61	2.99	0.1094
X_2_ X_3_	0.2236	2.68	0.1273	4.58	0.9212	0.3561	5.31	2.40	0.1470
X_2_ X_4_	1.70	20.43	0.0007	16.42	3.31	0.0941	50.36	22.78	0.0005
X_3_ X_4_	5.54	66.53	<0.0001	19.19	3.86	0.0730	28.30	12.81	0.0038
X_1_ X_1_	1.00	12.01	0.0047	51.18	10.30	0.0075	15.50	7.01	0.0213
X_2_ X_2_	0.1001	1.20	0.2946	69.92	14.07	0.0028	2.45	1.11	0.3132
X_3_ X_3_	1.56	18.71	0.0010	10.11	2.04	0.1792	6.05	2.74	0.1239
X_4_ X_4_	1.55	18.62	0.0010	94.74	19.07	0.0009	128.23	58.01	<0.0001
Residual	1.00			59.62			26.52		
Lack of Fit	0.8602	1.23	0.5291	54.72	2.23	0.3489	15.28	0.2719	0.9365
Pure error	0.1398			4.90			11.24		
Cor. total	23.46			1043.76			570.05		
R^2^	0.9574			0.9429			0.9535		
Adj. R^2^	0.9076			0.8762			0.8992		
Pred. R^2^	0.7684			0.6985			0.8107		
Adeq. Precision	18.66			15.19			13.92		
C.V. %	7.30			5.75			8.68		
	**FRAP**			**DPPH**			**Cyanin Chloride**		
	**SS**	**F-Value**	***p*-Value**	**SS**	**F-Value**	***p*-Value**	**SS**	**F-Value**	***p*-Value**
Model	2.43 × 10^5^	21.20	<0.0001	1.05 × 10^5^	31.39	<0.0001	3.86 × 10^6^	27.02	<0.0001
X_1_	10,040.96	12.29	0.0043	1225.33	5.13	0.0428	2469.16	0.24	0.6315
X_2_	39,400.87	48.22	<0.0001	1758.35	7.36	0.0189	2.28 × 10^5^	22.41	0.0005
X_3_	1473.65	1.80	0.2041	15,059.88	63.04	<0.0001	9.29 × 10^5^	91.08	<0.0001
X_4_	148.28	0.1815	0.6777	7.17	0.03	0.8654	2.602 × 10^5^	25.52	0.0003
X_1_ X_2_	14,662.44	17.94	0.0012	1233.55	5.16	0.0423	57,645.47	5.65	0.0349
X_1_ X_3_	1555.50	1.90	0.1928	7386.77	30.92	0.0001	2802.53	0.27	0.6096
X_1_ X_4_	37,957.63	46.45	<0.0001	7559.31	31.64	0.0001	58,116.76	5.70	0.0343
X_2_ X_3_	24,007.45	29.38	0.0002	27.53	0.12	0.7401	7.013 × 10^5^	68.79	<0.0001
X_2_ X_4_	1831.85	2.24	0.1601	13.84	0.06	0.8138	2133.24	0.21	0.6555
X_3_ X_4_	11,797.43	14.44	0.0025	129.72	0.54	0.4754	6482.65	0.64	0.4407
X_1_ X_1_	0.0004	5.34 × 10^7^	0.9994	26,500.12	110.92	<0.0001	1.382 × 10^5^	13.55	0.0031
X_2_ X_2_	10,571.53	12.94	0.0037	486.45	2.04	0.1791	8027.43	0.79	0.3923
X_3_ X_3_	2920.94	3.57	0.0830	7043.70	29.48	0.0002	1740.12	0.17	0.6868
X_4_ X_4_	55,583.85	68.03	<0.0001	16,268.41	68.09	<0.0001	1.358 × 10^6^	133.25	<0.0001
Residual	9805.10			2866.95			1.223 × 10^5^		
Lack of Fit	7166.32	0.5432	0.7914	2759.73	5.15	0.1735	1.129 × 10^5^	2.40	0.3300
Pure error	2638.78			107.22			9416.00		
Cor. total	2.524 × 10^5^			1.078 × 10^5^			3.979 × 10^6^		
R^2^	0.9611			0.9734			0.9693		
Adj. R^2^	0.9158			0.9424			0.9334		
Pred. R^2^	0.8086			0.8511			0.8309		
Adeq. Precision	19.43			22.77			21.88		
C.V. %	3.91			6.88			13.94		
	**Cyanidin-3-glucoside**			**Cyanidin-3-rutinoside**			**Pelargonidin-3-glucoside**		
	**SS**	**F-Value**	***p*-Value**	**SS**	**F-Value**	***p*-Value**	**SS**	**F-Value**	***p*-Value**
Model	6.03 × 10^6^	21.51	<0.0001	7.50 × 10^5^	24.74	<0.0001	1.06 × 10^6^	21.20	<0.0001
X_1_	89,142.53	4.45	0.0566	3137.70	1.45	0.2520	1.24 × 10^5^	34.50	<0.0001
X_2_	1.83 × 10^5^	9.14	0.0106	29,562.93	13.65	0.0031	136.08	0.0380	0.8487
X_3_	1.72 × 10^6^	85.62	<0.0001	5654.72	2.61	0.1321	2.07 × 10^5^	57.68	<0.0001
X_4_	9.16 × 10^5^	45.70	<0.0001	14,820.72	6,84	0.0226	34,969.07	9.77	0.0088
X_1_ X_2_	2.81 × 10^5^	14.00	0.0028	94,259.78	43.52	<0.0001	532.96	0.1489	0.7064
X_1_ X_3_	20,386.20	1.02	0.3330	12,004.45	5.54	0.0364	44,806.23	12.52	0.0041
X_1_ X_4_	5.532 × 10^5^	27.61	0.0002	71,727.02	33.12	<0.0001	6581.42	1.84	0.2001
X_2_ X_3_	28,332.30	1.41	0.2574	3.35 × 10^5^	154.77	<0.0001	2.04 × 10^5^	56.97	<0.0001
X_2_ X_4_	8081.15	0.40	0.5373	30,922.98	14.28	0.0026	39620.95	11.07	0.0060
X_3_ X_4_	5.30 × 10^5^	26.46	0.0002	1660.45	0.77	0.3985	1.00 × 10^5^	27.93	0.0002
X_1_ X_1_	4.00 × 10^5^	19.95	0.0008	7489.71	3.46	0.0876	1.40 × 10^5^	39.03	<0.0001
X_2_ X_2_	571.82	0.03	0.8687	3259.60	1.50	0.2434	24,047.83	6.72	0.0236
X_3_ X_3_	17,986.51	0.90	0.3621	1.19 × 10^5^	54.92	<0.0001	2.22 × 10^5^	62.11	<0.0001
X_4_ X_4_	1.11 × 10^6^	55.22	<0.0001	9929.23	4.58	0.0535	1.82 × 10^5^	50.94	<0.0001
Residual	2,40 × 10^5^			25,991.61			42,958.12		
Lack of Fit	2.07 × 10^5^	1.24	0.5262	23,826.04	2.20	0.3527	24,394.65	0.26	0.9409
Pure error	33,361.53			2165.57			18,563.47		
Cor. total	6.273 × 10^6^			7.76 × 10^5^			1.11 × 10^6^		
R^2^	0.9617			0.9665			0.9611		
Adj. R^2^	0.9170			0.9275			0.9158		
Pred. R^2^	0.7983			0.8192			0.8348		
Adeq. Precision	18.20			18.69			15.20		
C.V. %	15.84			12.87			16.64		

**Table 4:** Actual and coded values of independent variables.

Coded Values	Actual Values			
	X_1_	X_2_	X_3_	X_4_
−1.41	0.5	10	25	5
−1	2	20	40	15
0	3.5	30	60	25
+1	5	40	75	35
+1.41	6.5	50	90	45

X_1_ (Molar ratio); (water content, %); X_2_ (molar ratio) and X_3_ (temperature, °C); X_4_ (extraction time, min).

## Data Availability

Not applicable.

## Sample Availability

Samples of the compounds are available from the authors.
